# Supplementary material for: Rapid turnover of life-cycle-related genes in the brown algae
Source: Genome Biol. 2019 Feb 14;20:35. doi: 10.1186/s13059-019-1630-6 (PMC6374913; doi:10.1186/s13059-019-1630-6)
Supplement: Supplementary file 3 — Protein alignments, corresponding Gblocks html file and CDS sequences for the lineage conserved gametophyte-biased genes. (ZIP 2109 kb) [file 13059_2019_1630_MOESM3_ESM.zip › Con_GA_alignments/Ec-26_002830.aa_ali.fasta-gb.htm]

Ec-26\_002830.aa.fasta.trans.aa\_ali.fasta


## Gblocks 0.91b Results

Processed file: **Ec-26\_002830.aa.fasta.trans.aa\_ali.fasta**  
Number of sequences: **2**  
Alignment assumed to be: **Protein**  
New number of positions: **520** (selected positions are underlined in blue)

```
                         10        20        30        40        50        60
                 =========+=========+=========+=========+=========+=========+
Ec-26_002830     MLKQSRPSRTKGQRLPTLRAVGSSKTCSGEASANICSGQRRVSKRGRATDREHIAPEKSN
MSTRG.14807.1    ------------------------------------------------------------
                                                                             


                         70        80        90       100       110       120
                 =========+=========+=========+=========+=========+=========+
Ec-26_002830     GPSNDSHRFARNIPVEQERKIYGCNGSPMLDCGRGGRAEDGDEFTALSASPRSVNSADSF
MSTRG.14807.1    ------------------------------------------------------------
                                                                             


                        130       140       150       160       170       180
                 =========+=========+=========+=========+=========+=========+
Ec-26_002830     GTLASTGEESFSLELDGNANGSLAQEGEADAVGGNGTAEAGSASKGLGIVDRTLFDSWKE
MSTRG.14807.1    ------------------------------------------------------------
                                                                             


                        190       200       210       220       230       240
                 =========+=========+=========+=========+=========+=========+
Ec-26_002830     LPWQARLCPKAIEGQEVVRRASALEMENIRVQAWRRTQQLLATSAAREEEQRISGWWKGE
MSTRG.14807.1    ------------------------------------------------------------
                                                                             


                        250       260       270       280       290       300
                 =========+=========+=========+=========+=========+=========+
Ec-26_002830     AEKLWRIQSQRRQSHETLAARHEGELAAREDKRTELLSKGYGRELAKLDSMDEANHLESL
MSTRG.14807.1    ------------------------------------------------------------
                                                                             


                        310       320       330       340       350       360
                 =========+=========+=========+=========+=========+=========+
Ec-26_002830     RQAEMLFSFAVGDSSIAEETRKPTSPKEGVTTSTVAEGLDTVAAPGAYATPPGTTLRTSV
MSTRG.14807.1    ------------------------------------------------------------
                                                                             


                        370       380       390       400       410       420
                 =========+=========+=========+=========+=========+=========+
Ec-26_002830     RSPRVPDGREKEEGTLAGTKEQQQQQQEENGEARVVQETAEADCIEKISSHVQLELERVA
MSTRG.14807.1    ------------------------------------------------------------
                                                                             


                        430       440       450       460       470       480
                 =========+=========+=========+=========+=========+=========+
Ec-26_002830     LHESVMKATVDKFRRRRQSLAVILWCGLTAMYRDCRARARRTFVSECAADIGRIKQLASS
MSTRG.14807.1    ------------------------------------------------------------
                                                                             


                        490       500       510       520       530       540
                 =========+=========+=========+=========+=========+=========+
Ec-26_002830     PSSGGGSTGEGQNFPLSPPPPVPPPPAAGGTEDVLNEFCGSGSGTLSDCGESDSGITRPS
MSTRG.14807.1    ------------------------------------------------------------
                                                                             


                        550       560       570       580       590       600
                 =========+=========+=========+=========+=========+=========+
Ec-26_002830     GVDVADSPPDVQDALRKTTATTGGPGAAGHRERTRATRELRSEASRVARWDKLQRGVLRR
MSTRG.14807.1    ------------------------------------------------------------
                                                                             


                        610       620       630       640       650       660
                 =========+=========+=========+=========+=========+=========+
Ec-26_002830     STYEVTRLLTPDGLRRHDGGGWPNAWRQRGDRRGADGGVVESTLAVHKRARLSMRELRGR
MSTRG.14807.1    ------------------------------------------------------------
                                                                             


                        670       680       690       700       710       720
                 =========+=========+=========+=========+=========+=========+
Ec-26_002830     QADASLEARIIERSWMVSLAQWEAQLVKARAVAADAEVRELRILQREDAETARALEVSQS
MSTRG.14807.1    ------------------------------------------------------------
                                                                             


                        730       740       750       760       770       780
                 =========+=========+=========+=========+=========+=========+
Ec-26_002830     LARVIDREQNLVDDAQSSLVVDLAKIVADENAKRAEAAAVARRHWWAEARESLLDLFRSV
MSTRG.14807.1    ------------------------------------------------------------
                                                                             


                        790       800       810       820       830       840
                 =========+=========+=========+=========+=========+=========+
Ec-26_002830     HSSNWNDQESGGGCADVDVGIHGGGSATEKPAATSSASLQRIRTSLDELLRAEEILIEKA
MSTRG.14807.1    -------------------------------GGPLHLAAANIRRRIREAMEPCAKALEAA
                                                                          ###


                        850       860       870       880       890       900
                 =========+=========+=========+=========+=========+=========+
Ec-26_002830     ATDFLTAVNADECGGGGGGPLHVAAVENQRYTNEVLERCTKTFEAASFDPPTSTVRQPTP
MSTRG.14807.1    RDCLLSSAVEQEAQ------------------SEPENTVTKTHGTPSLAAGESDRRGRVR
                 ############                     ###########################


                        910       920       930       940       950       960
                 =========+=========+=========+=========+=========+=========+
Ec-26_002830     VNLNQWSTEGGSSSGHLGGAHNPEEELASFRGRLETDAVQLAVASFEAEVRQWAIDAAIP
MSTRG.14807.1    VSGGSDSGSRDGRRRHRGSNNAKEEDPDLFRIKLEADAIRLAAASYETEVRGLAIGTVFS
                 #######################################################     


                        970       980       990      1000      1010      1020
                 =========+=========+=========+=========+=========+=========+
Ec-26_002830     ETASEAVLPPCLAGAPSTMVEIEGKGSSPPFPLDGRPPSSDLTRSQLDLGRVARVKAFAR
MSTRG.14807.1    S-GTGATEPVALTALPAPAVGTEAR-DSPPLTQSDDPPASCLTRSELDLKRAARAGSYAR
                      ##################    #################################


                       1030      1040      1050      1060      1070      1080
                 =========+=========+=========+=========+=========+=========+
Ec-26_002830     TMEDMLSRTRKALLKETLQAATATENDYNLKVSSIISRHSGKISHLKTESRAKGDKALET
MSTRG.14807.1    ALGEMLFRARAALVKNALKAAAADEQTHDRGVTMVMYRHTRRAQDMRTRARVGGDEALRT
                 ############################################################


                       1090      1100      1110      1120      1130      1140
                 =========+=========+=========+=========+=========+=========+
Ec-26_002830     ALNEAETLSEERSWRSKTAAAIRRRALGPVVSWVGAAVDAELRRSERRRRRRQHRQHRIR
MSTRG.14807.1    ALKRAEVFHEERSSDWRTVAEMRRWVVGSVVRWIDGATRSHIQRSARRLRERQARQHRHA
                 ############################################################


                       1150      1160      1170      1180      1190      1200
                 =========+=========+=========+=========+=========+=========+
Ec-26_002830     ASLENFERNASEEQQDWLRHVRGRGHEATEVARTAISDHVAELILHERQRQQDQEGRYEA
MSTRG.14807.1    ASLRDSMRSISDEQGGLLYRVREEARDAIEAARGTIDGHVAELGHRRQEKEGARASAAAD
                 ###########################################                 


                       1210      1220      1230      1240      1250      1260
                 =========+=========+=========+=========+=========+=========+
Ec-26_002830     LSMAADT----------AKDLSSLQDCSSSAGEGTSTDDKDKPGEQGKMEQGEQ-LMALN
MSTRG.14807.1    VGLARDRQSFPRDGSSRAGDEPALREGVGDNGQGHEVPDGGEPRFAAFMTEEQAPNSAVE
                                  ################################        ###


                       1270      1280      1290      1300      1310      1320
                 =========+=========+=========+=========+=========+=========+
Ec-26_002830     QSALVMNTVAAAEGVNGDYVDDDHSG----GNFP-QPQFQGAKRQHVANQDGDGDVFVEP
MSTRG.14807.1    GSCRSKNTNEGDEDGGGDVQDVSGDEDNLRGTDPQENQDQGPYRSRAEEEDDKEDHGGNE
                 #####################                ##################     


                       1330      1340      1350      1360      1370      1380
                 =========+=========+=========+=========+=========+=========+
Ec-26_002830     PSLEG-LRVEAMAVLAAAEASCMCDIDAWVEEARRRELEMISAWMAEAASGDIEEREMTE
MSTRG.14807.1    TTVDVPAQREVLAAIAAAEASCLSRVFPWARDARHRARETAASWNEEVASGCRNERTTTN
                          ###################################################


                       1390      1400      1410      1420      1430      1440
                 =========+=========+=========+=========+=========+=========+
Ec-26_002830     TELETLESAFRAARKDADEAVSSACCEAYSLRPSGVSGAGAAPSGTEKTADPEPYATIGS
MSTRG.14807.1    TELETLALAFRAAKKEADEDVFGSHERDSRPRPPGVAGGHADLCPGEMSGSIN-WVPLSG
                 #####################          ################             


                       1450      1460      1470      1480      1490      1500
                 =========+=========+=========+=========+=========+=========+
Ec-26_002830     TETAAPGFLAEEEEECSALWPSLERPLSTQKLALKTIAAGSGGLCRDEKEGEEGKEAKLA
MSTRG.14807.1    VRAAAAGKQAESVPHC-----------------------------------EYRAEEQKE
                    #############                                   #########


                       1510      1520      1530      1540      1550      1560
                 =========+=========+=========+=========+=========+=========+
Ec-26_002830     ASRIITGLECELRAVVTDSFSSTGKVEEVRSMIRASSDELRVAFTLISEETQAKRKVRDL
MSTRG.14807.1    SSALWLSFERLLPSLQIDHQRRTGKGGSAE------------------------------
                 #########################                                   


                       1570      1580      1590      1600      1610      1620
                 =========+=========+=========+=========+=========+=========+
Ec-26_002830     ELVERARTEPEEAQEAKALAYGEHNQSPPATAAEGRVTDAERKNDVVPAISWEIAREDCG
MSTRG.14807.1    ------------------------------------------------------------
                                                                             


                 
                 =========
Ec-26_002830     APDGGIGTE
MSTRG.14807.1    -----CSYG
```

```
Parameters used
Minimum Number Of Sequences For A Conserved Position: 2
Minimum Number Of Sequences For A Flanking Position: 2
Maximum Number Of Contiguous Nonconserved Positions: 8
Minimum Length Of A Block: 10
Allowed Gap Positions: None
Use Similarity Matrices: Yes
```

```
Flank positions of the 11 selected block(s)
Flanks: [838  852]  [874  955]  [966  983]  [988  1183]  [1218  1249]  [1258  1281]  [1298  1315]  [1330  1401]  [1412  1427]  [1444  1456]  [1492  1525]  

New number of positions in Ec-26_002830.aa.fasta.trans.aa_ali.fasta-gb:  520  (31% of the original 1629 positions)
```
